# Rich diversity and potency of skin antioxidant peptides revealed a novel molecular basis for high-altitude adaptation of amphibians

**DOI:** 10.1038/srep19866

**Published:** 2016-01-27

**Authors:** Xinwang Yang, Ying Wang, Yue Zhang, Wen-Hui Lee, Yun Zhang

**Affiliations:** 1 Key Laboratory of Animal Models and Human Disease Mechanisms, Chinese Academy of Sciences & Yunnan Province, Kunming Institute of Zoology, Chinese Academy of Sciences, Kunming 650223, Yunnan, China; 2 Key Laboratory of Chemistry in Ethnic Medicine Resource, State Ethnic Affairs Commission & Ministry of Education, School of Ethnic Medicine, Yunnan Minzu University, Kunming 650500, China; 3 Department of Anatomy and Histology & Embryology, Faculty of Basic Medical Science, Kunming Medical University, Kunming 650500, China

## Abstract

Elucidating the mechanisms of high-altitude adaptation is an important research area in modern biology. To date, however, knowledge has been limited to the genetic mechanisms of adaptation to the lower oxygen and temperature levels prevalent at high altitudes, with adaptation to UV radiation largely neglected. Furthermore, few proteomic or peptidomic analyses of these factors have been performed. In this study, the molecular adaptation of high-altitude *Odorrana andersonii* and cavernicolous *O. wuchuanensis* to elevated UV radiation was investigated. Compared with *O. wuchuanensis*, *O. andersonii* exhibited greater diversity and free radical scavenging potentiality of skin antioxidant peptides to cope with UV radiation. This implied that *O. andersonii* evolved a much more complicated and powerful skin antioxidant peptide system to survive high-altitude UV levels. Our results provided valuable peptidomic clues for understanding the novel molecular basis for adaptation to high elevation habitats.

All organisms adapt to specific environments to ensure survival. Elucidating the molecular basis of adaptation is a fundamental and long-standing goal of modern evolutionary biology^1^,^2^. High-elevation (>2500 m) environments impose severe physiological challenges on organisms, particularly in regards to the reduced oxygen level, low temperature and elevated ultraviolet (UV) radiation^3^,^4^. Animals that have survived thousands of years in highlands have evolved adaptive mechanisms during their evolutionary history to cope with these harsh environmental stresses. The mechanisms of animal adaptation to high-altitude environments have been of considerable interest in recent years, and many genetic studies have been conducted on specific candidate genes, such as hemoglobin-related and mitochondrial genes^5^,^6^,^7^. Recent analysis of genome-wide variations among species and populations living in different altitudinal environments, including animal populations of yak^8^, Tibetan antelope^9^,^10^,^11^, snow leopard^12^ and wild boar^13^, human populations in the Andes^10^,^14^ and Ethiopia^15^,^16^, have begun to shed light on the genetic basis of many adaptive responses to low temperature and reduced oxygen at high altitudes. However, elevated UV radiation remains a neglected area of study. To date, genome-wide studies have only investigated endothermic species, with little information available on ectothermic species. Given the large physiological differences between such species, different mechanisms for adaptation to high-elevation environments are likely to have evolved. For example, instead of maintaining high metabolism for temperature stability, ectothermic species may lower their metabolic rate to cope with low external temperatures^4^. Current research on the molecular basis of high-altitude adaptation has primarily relied on genomic analysis, with comparative proteomic analyses, which are essential for understanding how animals adapt to high-elevation environments, still in their infancy. As yet, only a few related works have been conducted on plants^17^.

Amphibians, the first group of animals to form a link between terrestrial and aquatic environments, are widely distributed in a great variety of habitats worldwide. Amphibians living in high-altitude environments have evolved many behavioral and physiological adaptations to low temperatures and reduced oxygen at high altitudes^18^,^19^,^20^,^21^. For example, the adaptive significance of the alpha-chain chloride-binding sites of hemoglobin in response to low oxygen has been reported^22^. In addition, recent transcriptomic analysis of two amphibian species revealed that a set of candidate genes, including genes associated with oxygen binding, UV radiation response and free radical injury repair, may be related to high-elevation environments^23^. However, high-altitude adaptation in amphibians remains to be fully elucidated.

Skin is exposed to both endogenous and exogenous sources of oxidative stress and has developed various mechanisms to cope with increased oxidation^24^,^25^. One of the most important strategies is the modulation of the antioxidant system, which can be classified into two major groups: gene-encoded enzymes and non-gene-encoded low-molecular-weight antioxidants. The first group includes superoxide dismutase (SOD), catalase and peroxidase. The second group includes molecules such as glutathione (GSH) and vitamins C and E^24^,^26^. Radiation injury is considered one of the most significant non-biological stresses for amphibians because their skin is frequently exposed to a variety of harsh conditions; consequently, amphibian skin is endowed with an excellent chemical defense system composed of bioactive peptides, including a novel type of antioxidant peptides (AOPs) that was identified recently and characterized by gene-coded low-molecular weight peptides^27^,^28^,^29^. Amphibians living in plateau environments with strong UV radiation and a long duration of sunshine are at greater risk of radiation injury, and the identity and potential role of antioxidant peptides (AOPs) in high-altitude adaptation remains unclear.

*Odorrana andersonii* and *O. wuchuanensis* provide an excellent model system to study the adaptation of amphibian skin to high-altitude habitats. *Odorrana andersonii* lives in plateau areas (approximately 2500 m) of the Yunnan Province in China, which are characterized by a long duration of sunshine and strong UV radiation. *Odorrana wuchuanensis* is only distributed in a small number of caves (at an altitude of 800 m) in the Guizhou Province of China and encounter no light during their entire life cycle. Thus, we hypothesized that the AOPs of these two odorous frogs differ in amounts, structural characteristics and activities. Our results confirmed this hypothesis and provided valuable peptidomic clues for understanding the molecular basis of high-altitude adaptation in amphibians.

## Results

### Inducement of antioxidant activity of amphibian skin secretions by UV radiation

**Figure 1:**
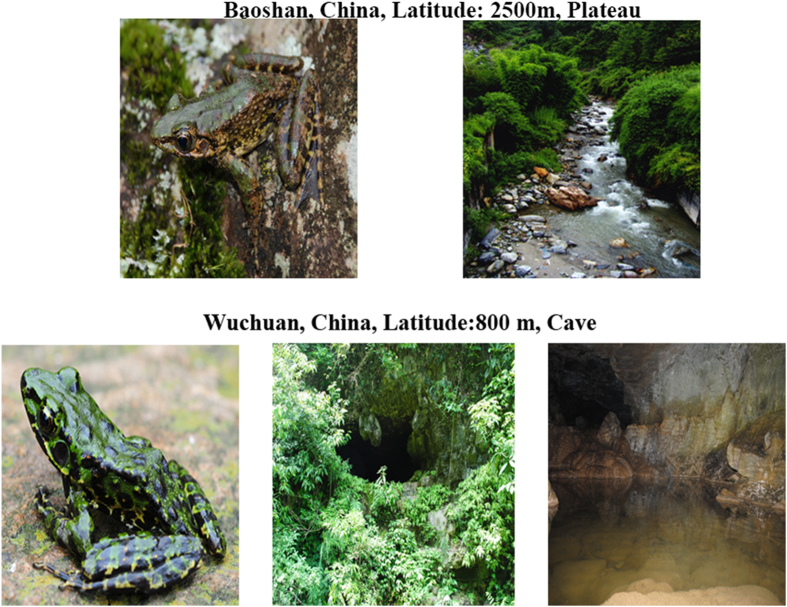
Distribution areas of *O. andersonii* and *O. wuchuanensis.* *Odorrana andersonii* is widely distributed on the Yun-Gui Plateau and was collected in Baoshan city in Yunnan Province, China. *Odorrana wuchuanensis* is only distributed in a few mountain caves in Guizhou Province.

**Figure 2:**
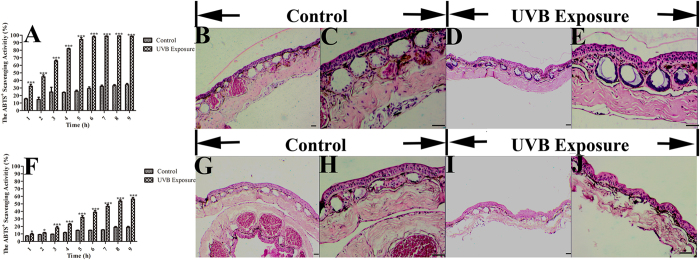
Response of *O. andersonii* and *O. wuchuanensis* skins to UVB radiation. Skins of *O. andersonii* and *O. wuchuanensis* were exposed to UVB radiation at a dose of 1600 μW/cm^2^ for 9 h. (**A**,**F**) show antioxidant activities in *O. andersonii* and *O. wuchuanensis* skin secretions induced by UVB exposure, respectively. The antioxidant activities were tested every 1 h. (**B**,**C**,**G**,**H**) show normal skin structures of *O. andersonii* and *O. wuchuanensis*, respectively. (**E**,**F**,**I**,**J**) show effects of UVB radiation on the skin structures of *O. andersonii* and *O. wuchuanensis*, respectively. *P < 0.01, ***P < 0.001. Bar = 250 μm. (**B**,**D**,**G**,**I**) are at 40×, (**C**,**E**,**H**,**J**) are at 100×.

As shown in Fig. 1, *O. andersonii* is widely distributed on the Yun-Gui Plateau in southwest China, whereas *O. wuchuanensis* is only distributed in a few mountain caves in Guizhou Province. To simulate the UV-radiated conditions of *O. andersonii* habitat, we exposed the skin of all frogs to UVB radiation (1600 μW/cm^2^) for 9 h. Compared with the dark environment, the antioxidant activities of the skin secretions from both species increased to different degrees following UVB radiation. The antioxidant activity of *O. andersonii* skin secretions increased after 1 h of UVB exposure and reached a maximum of ~99% after 5 h (Fig. 2A). For *O. wuchuanensis*, however, antioxidant activity did not exhibit an obvious increase until 3 h of UVB exposure and reached a maximum of only ~50% after 9 h (Fig. 2F). Thus, the antioxidant activities of both amphibian skin secretions were remarkably inducible by UV radiation, but the high-altitude *O. andersonii* skin secretions exhibited more potential antioxidant activates than cavernicolous *O. wuchuanensis*. These differences might be attributable to differences in the secretion, concentration or potency of the antioxidant substances induced by UVB radiation.

### Potential tolerance of *O. andersonii* and *O. wuchuanensis* skins to UV radiation

To characterize the damage to frog skin induced by UVB radiation, we performed HE staining to examine alterations in general frog skin morphology. Control skins not exposed to UVB radiation displayed a compact and stratified structure with normal epidermis, glands, dermis and blood vessels, with no evidence of inflammation or other damage (Fig. 2B,C,G,H). After direct UVB radiation, the skins of *O. andersonii* exhibited no obvious change (Fig. 2B,C), whereas the skins of *O. wuchuanensis* exhibited obvious tissue damage, including acute epidermal necrosis with inflammatory infiltrates in the superficial and deep dermis and destruction of glands (Fig. 2G,H). Antioxidants usually protect skin from impairment caused by UV radiation, and the different levels of damage to the skin of *O. andersonii* and *O. wuchuanensis* might be attributable to differences in the antioxidant activities of skin secretions. We speculated that the strong antioxidant activity of *O. andersonii* secretions protected their skin against damage induced by UV radiation.

### AOP diversity in *O. andersonii* and *O. wuchuanensis* skin secretions

**Figure 3:**
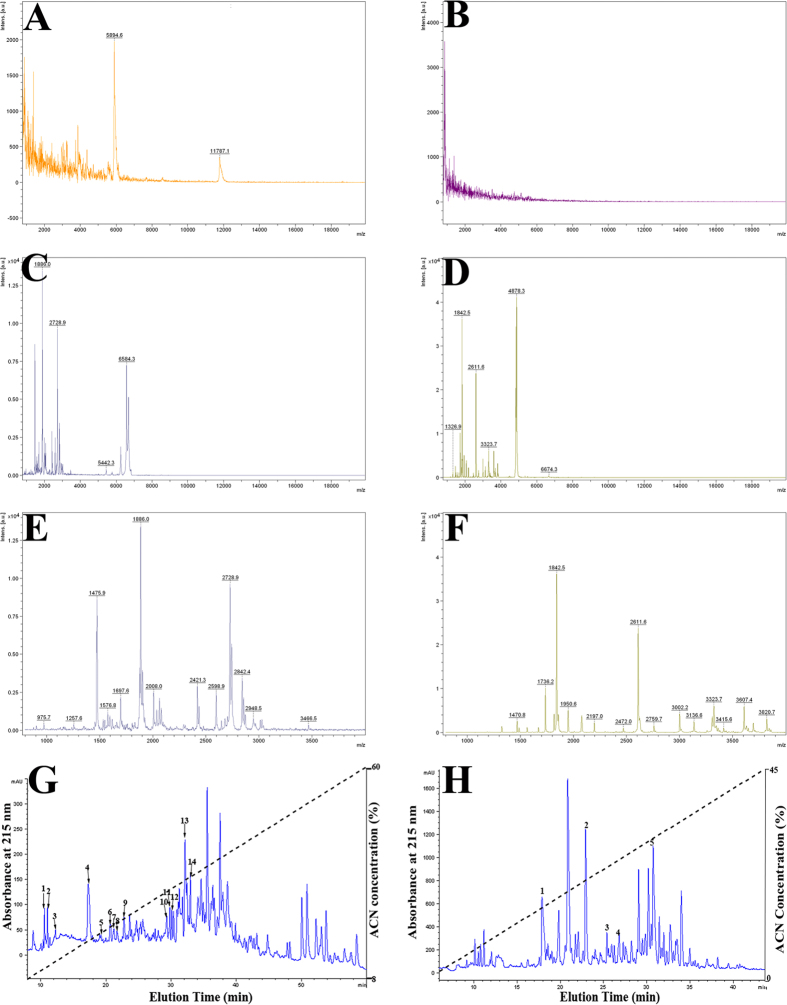
Overview of peptides induced by UV radiation. MS and HPLC analyses showed that UV radiation induced the secretion of many peptides compared with the controls. Left *O. andersonii* and right *O. wuchuanensis*. (**A**) MS analysis of *O. andersonii* skin secretions without UV exposure. (**B**) MS analysis of *O. wuchuanensis* skin secretions without UV exposure. (**C**) MS analysis of *O. andersonii* skin secretions with UV exposure, molecular weight range 800–20000 Da. (**D**) MS analysis of *O. wuchuanensis* skin secretions with UV exposure, molecular weight range 800–20000 Da. (**E**) MS analysis of *O. andersonii* skin secretions with UV exposure, molecular weight range 800–4000 Da. (**F**) MS analysis of *O. wuchuanensis* skin secretions with UV exposure, molecular weight range 800–4000 Da. (**G**) First RP-HPLC purification step of AOPs from skin secretions of *O. andersonii*. Skin secretions were applied to a C_18_ RP-HPLC column pre-equilibrated with 0.1% (v/v) TFA in water, and elution was achieved by a linear gradient (0–100% in 100 min) of 0.1% (v/v) TFA in ACN at a flow rate of 1 mL/min and was monitored at 215 nm. Fourteen peaks with ABTS^+^ scavenging activity were further purified. (**H**) First RP-HPLC purification step of AOPs from skin secretions of *O. wuchuanensis*. Skin secretions were applied to a C_18_ RP-HPLC column pre-equilibrated with 0.1% (v/v) TFA in water, and elution was achieved by a linear gradient (0–100% in 100 min) of 0.1% (v/v) TFA in ACN at a flow rate of 1 ml/min and was monitored at 215 nm. Five peaks with ABTS^+^ scavenging activity were further purified.

Under normal conditions without stimulation, the skins of both *O. andersonii* and *O. wuchuanensis* secreted few peptides or proteins (Fig. 3A,B). Following exposure to UVB radiation, however, an increase in peptide secretions was observed (Fig. 3C,D). Although molecular weights of these UVB-induced peptides were less than 4000 Da, remarkable differences in diversity were observed via mass spectrometry (MS) (Fig. 3E,F), which revealed that *O. andersonii* secreted a greater diversity of peptides (more than 100 molecules) than that of *O. wuchuanensis* (approximately 40 molecules). This difference in diversity was confirmed by initial HPLC analysis (Fig. 3G,H), which revealed more than 110 compounds in *O. andersonii* skin secretions but only 70 compounds in *O. wuchuanensis* skin secretions. The exact structures and functions of these peptides have not been investigated, but they likely play an important role in the scavenging of free radicals. Thus, in the present study, we characterized and compared peptides with the ability to scavenge free radicals.

**Figure 4:**
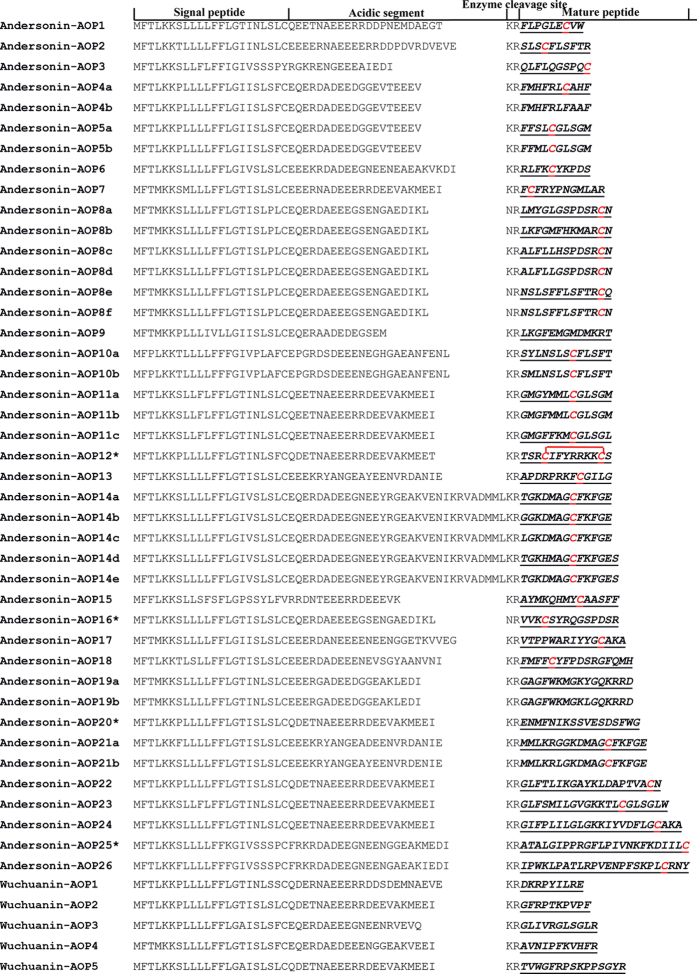
Structural diversity of AOPs. A total of 42 AOPs classified into 26 families were identified from O. andersonii. By contrary, only 5 AOPs were identified from O. wuchuanensis. All precursors of these identified AOPs shared a highly conserved motif, which was divided into four parts from the N-terminus to the C-terminus. The first part was an N-terminal hydrophobic signal peptide of about 22 residues in length, followed by an acidic segment that differed in length, ranging from 16–25 residues. The third part was a typical enzyme cleavage site ‘Lys-Arg’, sometimes mutated to ‘Val-Arg’, with the final part a C-terminal section of the mature peptide. The mature peptides confirmed by Edman degradation are underlined. ‘*’indicates that this peptide was identified and reported in our previous study^30^.

Identical purification procedures were used to identify AOPs in skin secretions induced by UVB exposure. After the first RP-HPLC purification step, 14 fractions with antioxidant activities were separated from high-altitude *O. andersonii* (Fig. 3G), but only 5 fractions were purified from cavernicolous *O. wuchuanensis* (Fig. 3H). Further purification enabled the isolation of 32 and 5 fractions with antioxidant activity and sufficient purity from the skin secretions of *O. andersonii* (Fig. S1) and *O. wuchuanensis* (Fig. S2), respectively. The complete amino acid sequences of these AOPs were presented in Fig. 4. After screening the skin cDNA library, another 10 molecules showing high sequence similarity were identified from *O. andersonii* AOPs, and were thus assumed to be AOPs. In total, 42 and 5 AOPs were identified by a combination of peptidomic and genomic methods from the skin secretions of *O. andersonii* and *O. wuchuanensis*, respectively (Fig. 4). The GenBank accession numbers assigned to the cDNA sequences encoding these AOPs are JX507085-JX507126.

Based on sequence similarity provided by BLASTp searches in NCBI, these identified AOPs from *O. andersonii* were grouped into 26 families named as Andersonin-AOP1 to Andersonin-AOP26 according to peptides’ length. Similarly, the AOPs from *O. wuchuanensis* were named as Wuchuanin-AOP1 to Wuchuanin-AOP5 (Fig. 4). Among these AOPs, we have previously reported Andersonin-AOP12, -AOP16 and -AOP20 and identified another eight peptides, including four from *O. andersonii* (Odorranain-A-OA12, Andersonin-G1, -H3 and -R1) and four from *O. wuchuanensis* (Wuchuanin-A1, -C1, -D1 and -E1), with obvious antioxidant activity^30^. In all, 46 AOPs classified into 30 different families were identified from high-altitude *O. andersonii*, whereas only nine AOPs grouped into 9 different families were recognized from cavernicolous *O. wuchuanensis*. These results indicated that the diversity of skin AOPs in high-altitude *O. andersonii* skins was much richer than that in cavernicolous *O. wuchuanensis*.

### Structural characteristics of AOPs from skins of *O. andersonii* and *O. wuchuanensis*

As shown in Fig. 4, all precursors of the identified AOPs shared a highly conserved motif that was divided into 4 parts from the N-terminus to the C-terminus. The first part was an N-terminal hydrophobic signal peptide approximately 22 residues in length, followed by an acidic segment that differed in length from 16–25 residues. The third part was a typical ‘Lys-Arg’ enzyme cleavage site, which was occasionally mutated to ‘Val-Arg’. The final part corresponded to the C-terminal section of the mature peptide.

As listed in Table S1, the molecular weights of the 37 AOPs determined by MS matched well with their theoretical molecular weights, indicating a lack of post-translational modification. Most of the AOP families contained only one member; however, some families exhibited great diversity, such as Andersonin-AOP8s and -AOP14s, which included six and five members, respectively (Fig. 3). The length of the AOPs also exhibited considerable diversity. Andersonin-AOP1 was the shortest AOP (9 amino acids), whereas Andersonin-AOP25 and -AOP26 were the longest AOP (24 amino acids, respectively).

Based on the number of cysteine residues, the identified AOPs were divided into three subfamilies: Family A, linear AOPs with no cysteine residue; Family B, linear AOPs with one free cysteine residue; and Family C, cyclic AOPs. Among the AOPs from *O. andersonii*, five and one belonged to Family A and C, respectively, and the remaining 36 belonged to Family B. This indicated that AOPs with one free cysteine were the dominant components of *O. andersonii*. Conversely, all AOPs from *O. wuchuanensis* belonged to Family A.

### Antioxidant activity of AOPs from *O. andersonii and O. wuchuanensis* skins

We also compared the antioxidant activities of AOPs from these two odorous frogs. Most *O. andersonii* skin AOPs exhibited potential ABTS^+^ scavenging activity (>90%), except for Andersonin-AOP9, -AOP13, -AOP17 and -AOP19a (Table 1). In comparison, except for Wuchuanin-AOP5, *O. wuchuanensis* AOPs displayed much weaker antioxidant activity. Moreover, most *O. andersonii* AOPs scavenged the free radical DPPH, whereas no peptides from *O. wuchuanensis* exhibited this activity.

All AOPs scavenged ABTS^+^ in a dose-dependent manner (Figs S3–S33). Andersonin-AOP1 exhibited the most potential ABTS^+^ scavenging activity and scavenged 99.280 ± 0.562% of ABTS^+^ at a concentration of 3 μM (n = 3, Fig. 5A). By contrast, Wuchuanin-AOP5, which exhibited the greatest ABTS^+^ scavenging potential among *O. wuchuanensis* AOPs, demonstrated 20-fold weaker antioxidant activity than that of Andersonin-AOP1 and required a concentration of approximately 60 μM to scavenge most of the ABTS^+^ (Fig. 5B). These results indicated that of AOPs from high-altitude *O. andersonii* exhibited more potential antioxidant activities than those of AOPs from cavernicolous *O. wuchuanensis*.

**Figure 5:**
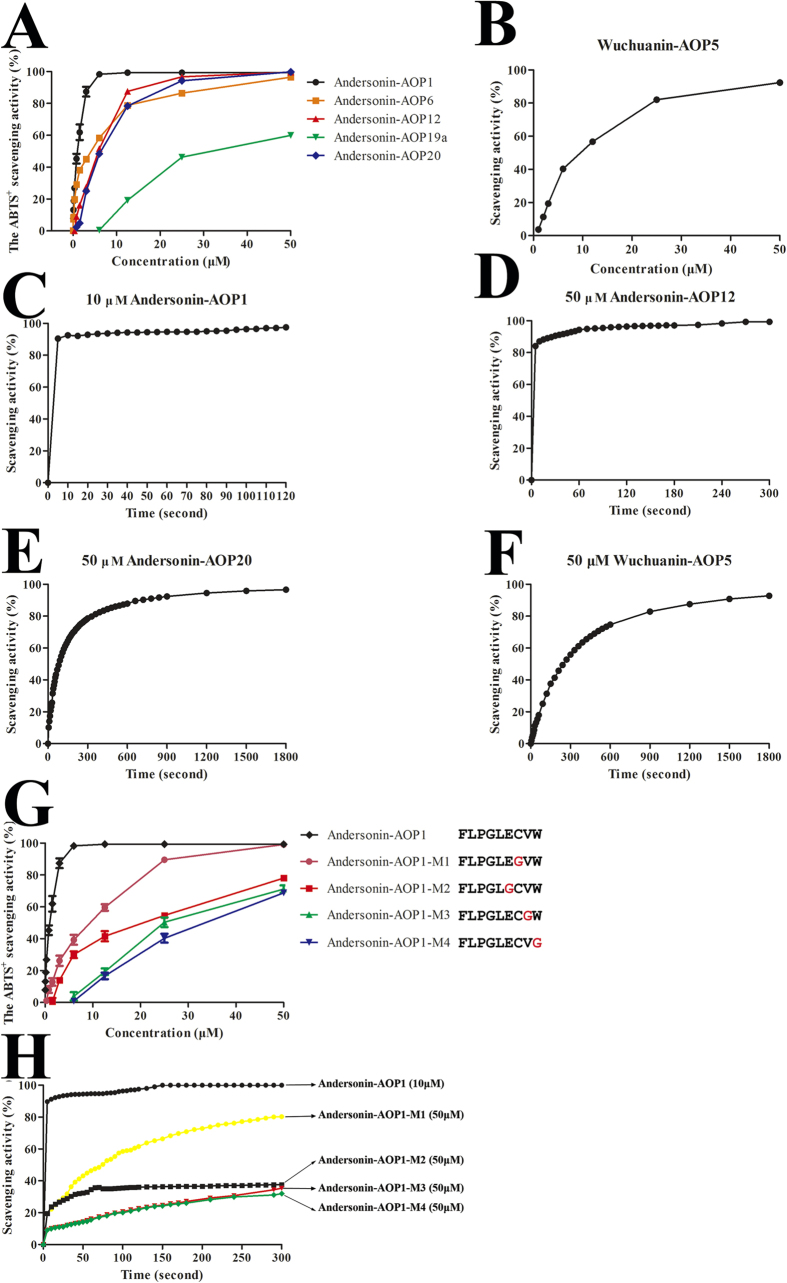
Antioxidant activities of different types of AOPs. (**A**) Antioxidant activities of AOPs from *O. andersonii*. Five AOPs representing different molecular types were selected to determine ABTS^+^ scavenging activity. Andersonin-AOP1 with free cysteine residue was the most powerful scavenger, while Andersonin-AOP19a, which had no cysteine residue, was the weakest scavenger. (**B**) Antioxidant activities of AOPs from *O. wuchuanensis*. Wuchuanin-AOP5 was the most powerful antioxidant; however, its activity was much weaker than that of Andersonin-AOP1. (**C**–**F**) Andersonin-AOP1 (representative of AOPs with one free cysteine) and Andersonin-AOP12 (representative of AOPs with one disulfide bridge) exerted high ABTS^+^ scavenging efficiency (**A**,**B**). In contrast, Andersonin-AOP20 and Wuchuanin-AOP5 (representative of AOPs without cysteine) showed comparatively low scavenging efficiency. Data are mean values of three independent experiments performed in triplicate. (**G**,**H**) Four mutants of Andersonin-AOP1 were designed. The replacement of the last four residues resulted in a decrease in ABTS^+^ scavenging ability, and the replacement of free cysteine lead to a notable decrease in ABTS^+^ scavenging efficiency.

We also compared the scavenging rate of Andesonin-AOP1 and Wuchuannian-AOP5. As shown in Fig. 5C,D, Andersonin-AOP1 reached the maximum scavenging rate in less than 5 s; however, Wuchuanin-AOP5 exhibited a 600-fold slower scavenging rate and required more than 1800 s to reach its maximum rate. These results indicated that the free radical scavenging rate of the tested AOP from high-altitude *O. andersonii* was much faster than that of the AOP from cavernicolous *O. wuchuanensis*.

### Role of free cysteine in free radical scavenging activities of amphibian skin AOPs

Most AOPs from *O. andersonii* contained a free cysteine residue, while all AOPs from *O. wuchuanensis* contained no cysteine residue (Fig. 4). Based on this observation, it might be reasonable to speculate that free cysteine plays an important role in the AOPs system of *O. andersonii*. Accordingly, we selected five different AOPs from *O. andersonii*, Andersonin-AOP19a and -AOP20 (no Cys), -AOP1 and -AOP6 (one Cys) and -AOP12 (two linked Cys) to compare their antioxidant activities. At a concentration of 3 μM, Andersonin-AOP1 scavenged almost all the ABTS^+^; however, Andersonin-AOP6 needed a concentration of 50 μM to exhibit the same potentiality. When the concentration was lower than 12 μM, the antioxidant activity of Andersonin-AOP12 was lower than that of -AOP6; however, when the concentration was higher than 12 μM, the opposite result was found. The same situation also occurred for Anersonin-AOP20. Among these five AOPs, Andersonin-AOP19a was the worst ABTS^+^ scavenger (Fig. 5A). At low concentrations, AOPs with a free cysteine residue showed much stronger antioxidant activities, although the difference was not so obvious at high concentrations. Thus we speculated that the cysteine residue did not determine the antioxidant activities of AOPs and the antioxidant potentiality of specific AOPs did not rely on free cysteine.

We also investigated whether cysteine had an influence on the scavenging rate of AOPs. Andesonin-AOP1 and Andersoin-AOP12 reached their maximum scavenging rates within 5 s (Fig. 5C,D); however, AOPs with no cysteine residue, including Andersonin-AOP20 and Wuchuanin-AOP5, required approximately 1800 s to reach their maximum scavenging rates (Fig. 5E,F). These results implied that cysteine played an important role in the scavenging rates of AOPs. To test this speculation, we selected Andersonin-AOP1 as a template and designed four mutants, Andersonin-AOP1-M1 (C7/G7), Andersonin-AOP1-M2 (E6/G6), Andersonin-AOP1-M3 (V8/G8) and Andersonin-AOP1-M4 (W9/G9). As illustrated in Fig. 5G, the replacement of these four residues of Andersonin-AOP1 decreased its free radical scavenging activity and the ninth residue of W and eighth residue of V notably decreased the ABTS^+^ scavenging activity. We also investigated the role of these four residues on ABTS^+^ scavenging efficiency of Andersonin-AOP1. As illustrated in Fig. 5H, -AOP1 scavenged more than 90% of ABTS^+^ within 5 s, while -M2, -M3 and -M4 achieved maximum scavenging rates within 10 s. However, the efficiency of -M1 was much slower, requiring more than 300 s to achieve its maximum rate. These results indicated that free cysteine was responsible for the ABTS^+^ scavenging efficiency of -AOP1.

## Discussion

High-elevation environments impose severe physiological challenges on organisms, and elucidating the process and molecular basis of adaptation is a long-standing goal of modern evolutionary biology^1^,^2^. Despite extensive research, however, knowledge of these crucial issues has been limited to genomic studies of adaptations of endothermic species to the lower oxygen and temperature levels at high elevation, with comparative proteomic analyses, which are essential for understanding how animals adapt to high-elevation environments, still in their infancy. In our previous work, we explored the bioactive peptides of amphibian skins for the development of novel medicines and in the hope of providing meaningful clues on how they adapt to specific surroundings. For example, because bare amphibian skins are vulnerable to pathogens, a variety of antimicrobial peptides are secreted to kill microbes directly^31^,^32^,^33^,^34^,^35^,^36^,^37^, and protein weapons have evolved to cope with infection^38^. Following these studies, especially those on the antimicrobial peptides of odorous frogs in which several AOPs were cloned^30^, we chose high-altitude *O. andersonii* and cavernicolous *O. wuchuanensis* for peptidomic investigation to understand the molecular basis for ectothermic species adaptation to UV radiation at high altitude. Although they belong to the same genus (Odorrana, Ranidae), differences in their habitat are remarkable. *Odorrana andersonii* is widely distributed in the Yun-Gui Plateau in southwestern China, and though its nocturnal lifestyle does limit its exposure to UV radiation, it is still at great risk of direct exposure throughout its lifecycle and during certain activities. Conversely, *O. wuchuanensis*, which is only distributed in a few mountain caves in Guizhou Province, experiences no UV radiation throughout its entire life cycle (Fig. 1).

The antioxidant activity of *O. andersonii* and *O. wuchuanensis* skin secretions increased slightly when the frogs were maintained in a dark environment without UV radiation (Fig. 2), which may be due to amphibian skins being susceptible to biological or non-biological injuries that induce oxidative stress, such as microorganism infection, parasitization, predation, radiation and aseptic wounds^27^,^39^. When mammals are exposed to UVB radiation, for example, an increase in oxidative stress and levels of antioxidant substances, such as SOD and GSH, is often observed^40^. However, how amphibian skin responds when directly exposed to UV radiation remains unclear.

We demonstrated that antioxidant activities were inducible for both selected frogs; however, skin reactions to UV radiation were much stronger in *O. andersonii* than that in *O. wuchuanensis*, as evidenced by the differences in their free radical scavenging activities. We also evaluated damage to the skin tissue induced by UV radiation. Interestingly, under the same dose of UV radiation, the skin tissue of *O. andersonii* was nearly undamaged, whereas the skin of *O. wuchuanensis* exhibited severe necrosis (Fig. 2), indicating that *O. andersonii* skin was much more tolerant to high-altitude UV radiation. Because antioxidants usually protect skin from damage induced by UV radiation, we speculated that differences in the antioxidant activities of skin secretions were responsible for the different levels of damage induced by UV radiation.

Skins of *O. andersonii* secreted many more peptides than that of *O. wuchuanensis* when exposed to UV radiation. While no information was available on the structures and functions of these peptides, their molecular weights were less than 4000 Da (Fig. 3 and Table S1) and might function as antioxidants. We identified 46 AOPs from *O. andersonii*, but only nine from *O. wuchuanensis*, which indicated that the diversity of AOPs identified from *O. andersonii* skin secretions was much richer than that of AOPs from *O. wuchuanensis* skin secretions. Furthermore, antioxidant activities of *O. andersonii* AOPs were more potent than those of *O. wuchuanensis* (Table 1, Fig. 5 and S3–33). These results revealed that *O. andersonii* skin AOPs were more potent and numerous under UV radiation challenge than those of *O. wuchuanensis*. Thirty-six AOPs were previously identified from the skin secretions of *Rana pleuraden*, a pond frog widely distributed on the Yun-Gui Plateau^28^. The AOPs from *O. andersonii* and *R. pleuraden* exhibited similar diversities and antioxidant potencies but differed in their primary structures. Thus, powerful AOPs may be a characteristic of amphibians from plateau environments. Although antioxidant enzymes such as SOD increase with altitude in some plant species^17^, their profiles of expression in animals remain scare and to be elucidated.

We found that free cysteine did not impact antioxidant activities, but did have considerable influence on the scavenging rate of AOPs (Fig. 5). Generally speaking, AOPs containing free cysteine exhibited a much faster free radical scavenging rate. In a previous report, free cysteine was proven to be crucial in amphibian AOPs^29^. Most AOPs from *O. andersonii* skin contained a free cysteine (Fig. 4), this notable and interesting feature also implied that *O. andersonii* skin AOPs were more potent than those of *O. wuchuanensis*.

A potential issue to be considered is that, in addition to the absence of UV exposure, *O. wuchuanensis* also lives in an obscured environment with high humidity and low temperature. Thus, the molecular basis of amphibian adaptation to high-altitude or cave habitats is likely comprehensive and closely interrelated. This research eliminated these factors by use of a simulated environment and directly demonstrated that AOPs were induced by UV exposure in both frog species. Our results suggested that *O. andersonii* skin evolved a much more complicated and powerful AOPs system. However, we cannot exclude the possibility that these peptides also function in obscured habitats with high humidity and low temperatures, and further research is warranted to characterize the functions of these peptides under different conditions.

## Conclusions

Our results showed that the skins of high-altitude *O. andersonii* were much more tolerant to UV radiation than that of *O. wuchuanensis*, and demonstrated much richer AOP diversity and greater potency of free racial scavenging activities. These findings indicated that to survive in environments with elevated UV radiation, the skins of *O. andersonii* evolved a much more complicated and powerful antioxidant peptide system. Our results also provided the first peptidomic clues to elucidate the unique molecular basis of adaptation to high altitudes in amphibians.

## Materials and Methods

### Animals and ethics statement

*Odorrana andersonii* specimens were collected in Baoshan City, Yunnan Province, China, and *O. wuchuanensis* specimens were collected in Wuchuan County, Guizhou Province, China. All study protocols and procedures were approved by the Ethics Committee of the Institutional Review Board of the Kunming Institute of Zoology, Chinese Academy of Sciences, and were conducted in strict accordance with the guidelines for Animal Care and Use at the Kunming Institute of Zoology.

### Inducibility of antioxidant activities of skin secretions and skin tolerance to UVB exposure

Once at the laboratory, the odorous frogs were maintained in an artificial pond with food sufficient for one week. Three adult frog specimens of each species were weighed (50–52 g), washed with ddH_2_O and transferred to a plastic box (13 × 9 × 9 cm) filled with 100 ml of ddH_2_O either in the dark (negative control) or in a UVB-exposed environment for 9 h at room temperature; the seclusion, humidity and other parameters of the artificial environments were identical. The UVB radiation dose was 1600 μW/cm^2^, equivalent to midday in summer in Baoshan city. To confirm the inducibility of the antioxidant activities of the skin secretions, liquid skin secretion samples (500 μl) were collected every 1 h to test their free radical scavenging activities. To compare the extent of damage to skin tissues, the frogs were sacrificed, and the dorsum skins were isolated rapidly for HE staining after UVB radiation exposure.

### Collection of skin secretions

*Odorrana andersonii* and *O. wuchuanensis* were exposed to UVB at 1600 μW/cm^2^ for 9 h in a plastic box (13 × 9 × 9 cm) filled with 100 ml of ddH_2_O. The collected solutions were centrifuged at 4000 rpm for 15 min, and the supernatants were lyophilized and stored at −80 °C until use.

### Peptide purification

Peptides were purified in two steps by reverse-phase (RP) chromatography. The dissolved samples were applied to a C_18_ RP-HPLC column (Hypersil BDS C_18_, 4.0 × 300 mm, Elite, Dalian, China) pre-equilibrated in 0.1% (v/v) trifluoroacetic acid (TFA) in water. The peptides were eluted by a linear gradient (0–100% in 100 min) of 0.1% (v/v) TFA in acetonitrile (ACN) at a flow rate of 1 ml/min and monitoring at 215 nm. Peaks of antioxidant activity were collected and lyophilized, and these samples were then further purified on the same C_18_ column with elution at a more shallow gradient of 0.1% (v/v) TFA in ACN (0–100% in 200 min).

### Structural analysis of peptides

The observed molecular weights and purities of the samples were determined using an autoflex speed TOF/TOF mass spectrometer (Bruker Daltonik GmbH, Bremen, Germany) in linear mode with α-cyano-4-hydrorycinnamic acid as the matrix. All procedures were conducted according to the manufacturer’s standard protocols, and the data were analyzed using the software package provided by the manufacturer.

The complete peptide sequences were determined by Edman degradation on a PPSQ-31A protein sequencer (Shimadzu, Japan) according to the manufacturer’s protocols.

### Construction of the skin cDNA library

Total RNA was extracted using TRIzol Reagent (Invitrogen, Carlsbad, CA, USA) from the skin of one individual from each frog species. The frogs were washed with deionized water and sacrificed, with the skins immediately stripped, cut into pieces and ground in liquid nitrogen. We purified the mRNA using an Absolutely mRNA Purification Kit according to standard protocols (Stratagene, Canada). The cDNA was synthesized using SMART^TM^ techniques with a SMART cDNA Library Construction Kit (Clontech, Canada). The first strand of cDNA was synthesized using 3′ SMART CDS III/3′ PCR primer, SMART IV oligonucleotide and SMART^TM^ MMLV Reverse Transcriptase (Clontech, Canada). The second strand was amplified using Advantage polymerase with 5′ PCR primer and CDS III/3′ PCR primer according to the protocols of the kit.

### Screening of cDNAs encoding AOP precursors

The synthesized cDNAs were used as templates for high-stringency PCR amplification to screen for cDNAs encoding AOP precursors. Two primers, S1 (5′-CCAAA(G/C)ATGTTCACC(T/A)TGAAGAAA-3′) and different 3′ PCR primers designed according to the peptide sequences provided by Edman degradation were used in the PCR reactions. Advantage polymerase was used in the reactions. The PCR procedure was as follows: 2 min at 94 °C, followed by 30 cycles of 10 s at 92 °C, 30 s at 50 °C and 40 s at 72 °C. The PCR products were then recovered using a DNA Gel Extraction Kit (Bioteke, China) and ligated into the vector pMD19-T (Takara Biotechnology, China). Finally, the PCR products were cloned into competent *Escherichia coli* DH5α. Clones were randomly selected for DNA sequencing on an Applied Biosystems DNA sequencer (ABI 3730XL, USA).

### Peptide synthesis

All peptides used in the evaluations of bioactivity were synthesized by solid-phase synthesis on an Applied Biosystems model 433A peptide synthesizer according to standard protocols, as previously reported^32^. Purities of the synthetic peptides were greater than 95%.

### Free radical scavenging activity assay

Two free radical scavenging tests were adopted to evaluate the antioxidant activity of the samples.

A 2, 2′-azino-bis (3-ethylbenzothiazoline-6-sulfonic acid) (ABTS) scavenging test was performed as previously described^41^, with some modification. Briefly, a stock solution of ABTS radical (Sigma-Aldrich, USA) was prepared by incubating 2.8 mM potassium persulfate (Sigma-Aldrich, USA) with 7 mM ABTS in water for at least 6 h in the dark, after which it was used immediately. The stock solution was diluted 50-fold with ddH_2_O. The samples dissolved in water were added, and the same volume of solvent was used as a negative control. Vitamin C dissolved in H_2_O was used as the positive control. The reaction was kept from light for 10 min. The decrease in absorbance at 415 nm indicated the antioxidant activity of the samples. The rate of free radical scavenging (%) was calculated by (A_blank_–A_sample_) × 100/A_blank_.

A 2, 2-diphenyl-1-picrylhydrazyl (DPPH) scavenging test was also performed as previously described^42^, with some modification. Briefly, the assay mixture contained 190 μl of 5 × 10^−5^ M DPPH radical (Sigma-Aldrich, St Louis, MO, USA) dissolved in methanol. The sample solution (10 μl) was then incubated in a sealed 1.5-ml microcentrifuge tube for 30 min at room temperature, and the absorbance was read against a blank at 517 nm. The DPPH scavenging activity (%) was calculated by (A_blank_–A_sample_) × 100/A_blank_.

### Hematoxylin-eosin (HE) staining

Isolated frog skin tissues were fixed in 10% buffered formalin overnight and then immersed in 70% ethanol for storage at room temperature. The tissues were embedded in paraffin. Sections with a thickness of 5 μm were cut on positively charged slides. The sections were stained with HE solution. All tissue samples were examined and photographed in a blinded manner. Images were captured using an Olympus BX51 microscope (Olympus, Tokyo, Japan) at ×40 or ×100 magnification^43^.

## Additional Information

**How to cite this article**: Yang, X. *et al.* Rich diversity and potency of skin antioxidant peptides revealed a novel molecular basis for high-altitude adaptation of amphibians. *Sci. Rep.* **6**, 19866; doi: 10.1038/srep19866 (2016).

## Supplementary Material

Supplementary Data

## Figures and Tables

**Table 1:** Antioxidant activities of AOPs from the skin of odorous frogs.

Samples	Free radical scavenging activity (%)		
	ABTS^+^	Figure	DPPH
H_2_O	ND		ND
Vitamin C	98.52 ± 1.34		93.97 ± 1.49
Andersonin-AOP1	99.28 ± 0.56	5A	ND
Andersonin-AOP2	98.95 ± 1.00	S3	89.39 ± 2.31
Andersonin-AOP3	98.98 ± 0.95	S4	64.29 ± 1.43
Andersonin-AOP4a	91.80 ± 1.79	S5	69.36 ± 1.37
Andersonin-AOP5a	99.70 ± 0.44	S6	71.90 ± 0.96
Andersonin-AOP6	96.46 ± 0.69	5A	ND
Andersonin-AOP7	99.07 ± 0.85	S7	79.88 ± 1.29
Andersonin-AOP8a	99.61 ± 0.17	S8	55.78 ± 0.98
Andersonin-AOP8b	97.61 ± 1.84	S9	72.36 ± 2.01
Andersonin-AOP8c	90.61 ± 1.89	S10	61.11 ± 1.46
Andersonin-AOP8e	99.28 ± 0.62	S11	78.21 ± 2.75
Andersonin-AOP9	51.33 ± 1.63	S12	11.90 ± 2.39
Andersonin-AOP10a	99.70 ± 0.44	S13	87.32 ± 0.35
Andersonin-AOP11a	99.47 ± 0.57	S14	90.23 ± 4.59
Andersonin-AOP11c	94.80 ± 1.05	S15	65.38 ± 3.12
Andersonin-AOP12	99.61 ± 0.44	5A	75.24 ± 2.52
Andersonin-AOP13	84.69 ± 0.58	S16	33.02 ± 1.45
Andersonin-AOP14a	99.64 ± 0.55	S17	67.55 ± 1.58
Andersonin-AOP14b	98.31 ± 1.13	S18	70.49 ± 2.51
Andersonin-AOP14d	99.30 ± 1.13	S19	85.32 ± 3.78
Andersonin-AOP15	99.04 ± 1.57	S20	91.01 ± 4.37
Andersonin-AOP16	98.32 ± 1.35	S21	86.19 ± 1.72
Andersonin-AOP17	66.67 ± 1.81	S22	35.24 ± 2.08
Andersonin-AOP18	99.16 ± 1.45	S23	40.23 ± 2.98
Andersonin-AOP19a	60.00 ± 1.92	5A	ND
Andersonin-AOP20	99.82 ± 0.09	5A	ND
Andersonin-AOP21a	94.69 ± 0.58	S24	57.98 ± 4.92
Andersonin-AOP22	99.25 ± 1.28	S25	60.79 ± 1.53
Andersonin-AOP23	97.95 ± 2.15	S26	94.38 ± 0.50
Andersonin-AOP24	99.96 ± 0.06	S27	ND
Andersonin-AOP25	95.74 ± 2.33	S28	77.41 ± 0.87
Andersonin-AOP26	80.77 ± 4.36	S29	41.27 ± 5.09
Wuchuanin-AOP1	33.12 ± 1.23	S30	ND
Wuchuanin-AOP2	13.26 ± 0.51	S31	ND
Wuchuanin-AOP3	17.21 ± 1.01	S32	ND
Wuchuanin-AOP4	11.89 ± 0.89	S33	ND
Wuchuanin-AOP5	92.34 ± 2.31	5B	ND

All AOPs were evaluated at a concentration of 50 μM. H_2_O and vitamin C were used as negative and positive controls, respectively. ‘ND’ indicates ‘Not Detectable’. Data are mean values of three independent experiments performed in triplicate.
