# Supplementary material for: Rich diversity and potency of skin antioxidant peptides revealed a novel molecular basis for high-altitude adaptation of amphibians
Source: Sci Rep. 2016 Jan 27;6:19866. doi: 10.1038/srep19866 (PMC4728494; doi:10.1038/srep19866)
Supplement: Supplementary Data [file srep19866-s1.pdf]

Rich diversity and potency of skin antioxidant peptides revealed a novel molecular basis for high-altitude adaptation of amphibians

Xinwang Yang, Ying Wang, Yue Zhang, Wen-Hui Lee, Yun Zhang

Table S1. Peptidomic analysis of AOPs purified from *O. andersonii and O. wuchuanensis* skin secretions

Samples	TMW[a] Da	OMW[b] Da	Difference Da
Andersonin-AOP1	1063.28	1063.6	-0.32
Andersonin-AOP2	1160.35	1160.3	0.05
Andersonin-AOP3	1120.29	1120	0.29
Andersonin-AOP4a	1308.58	1308.3	0.28
Andersonin-AOP5a	1061.28	1060.8	0.48
Andersonin-AOP6	1256.49	1256.6	-0.11
Andersonin-AOP7	1474.5	1474.7	-0.2
Andersonin-AOP8a	1412.6	1412.5	0.1
Andersonin-AOP8b	1582.96	1582.9	0.06
Andersonin-AOP8c	1472.68	1472.2	0.48
Andersonin-AOP8e	1549.76	1549.9	-0.14
Andersonin-AOP9	1543.88	1543.7	0.18
Andersonin-AOP10a	1481.68	1481.4	0.28
Andersonin-AOP11a	1350.71	1350.2	0.51
Andersonin-AOP11c	1347.67	1347.4	0.27
Andersonin-AOP12	1645.98	1646	-0.02
Andersonin-AOP13	1429.7	1429.8	-0.1
Andersonin-AOP14a	1390.59	1390.9	-0.31
Andersonin-AOP14b	1346.54	1346.2	0.34
Andersonin-AOP14d	1499.72	1499.7	0.02
Andersonin-AOP15	1698	1698	0
Andersonin-AOP16	1581.77	1581.9	-0.13
Andersonin-AOP17	1696	1695.9	0.1
Andersonin-AOP18	2060.39	2061.2	-0.81
Andersonin-AOP19a	1885.18	1884.8	0.38
Andersonin-AOP20	1977.13	1977.1	0.03
Andersonin-AOP21a	2006.44	2007.1	-0.66
Andersonin-AOP22	2095.48	2095.3	0.18
Andersonin-AOP23	2180.69	2179.5	1.19
Andersonin-AOP24	2437.02	2437.6	-0.58
Andersonin-AOP25	2597.2	2596.5	0.7
Andersonin-AOP26	2840.38	2839.4	0.98
Wuchuanin-AOP1	1189.38	1190.3	0.92
Wuchuanin-AOP2	1145.37	1145.3	0.07
Wuchuanin-AOP3	1140.39	1140.6	0.21
Wuchuanin-AOP4	1327.6	1328.4	0.6
Wuchuanin-AOP5	1734.98	1736	1.02

The 32 peaks from the HPLC procedure were taken to determine average molecular weight, together with purity, as shown in Figs. S1 to S32. The complete sequences of native AOPs with good purity were then analyzed by Edman degradation and the detailed sequences are shown in Fig. 2.

a. TMW, theoretical average molecular weight calculated at Peptide Mass

b. OMW, observed average molecular weight achieved by mass-spectrometry analysis

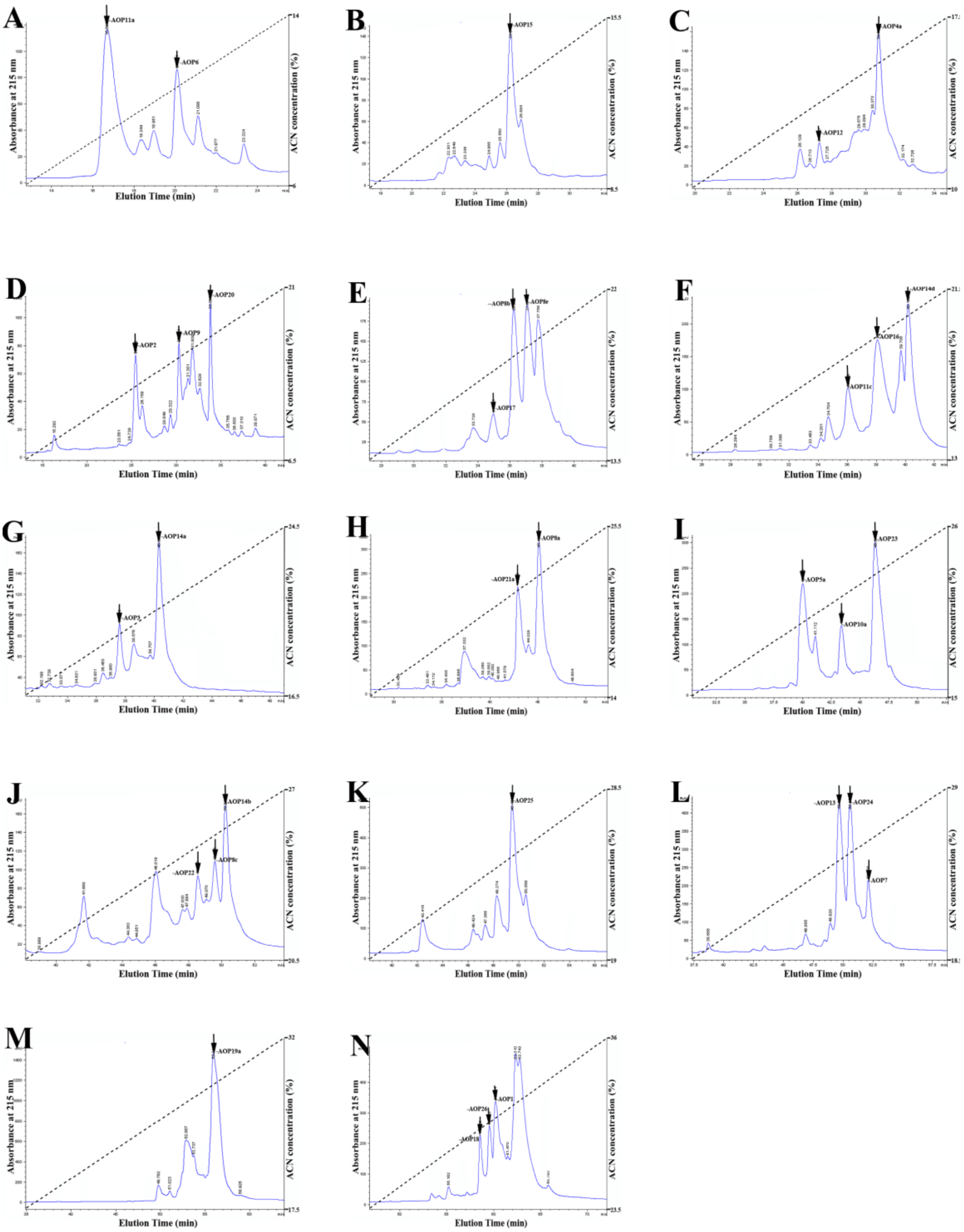

Figure S1. The second RP-HPLC purification of AOPs from skin secretions of *O. andersonii*

A-N showed the 14 peaks from first RP-HPLC (Fig. 3G) purification procedure were applied to a C_{18} RP-HPLC column pre-equilibrated with 0.1% (v/v) TFA in water, and elution was achieved by a linear gradient (0-100% in 200 min) of 0.1% (v/v) TFA in ACN at a flow rate of 1 mL/min and was monitored at 215 nm. The identified AOPs were named as Andersonin-AOPs and showed by an arrow.

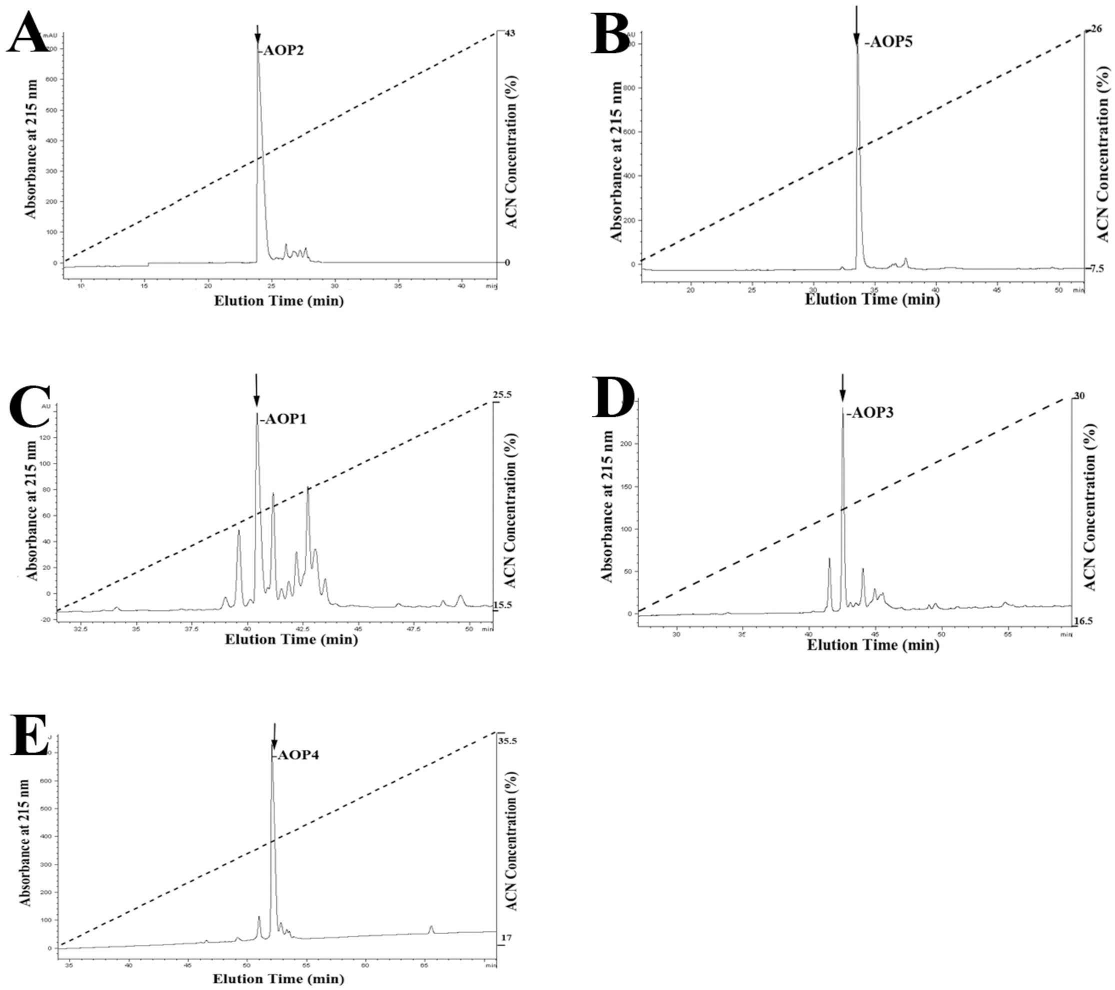

Figure S2. The second RP-HPLC purification of AOPs from skin secretions of *O. wuchuanensis*

A-E showed the 5 peaks from first RP-HPLC (Fig. 3H) purification procedure were applied to a C_{18} RP-HPLC column pre-equilibrated with 0.1% (v/v) TFA in water, and elution was achieved by a linear gradient (0-100% in 200 min) of 0.1% (v/v) TFA in ACN at a flow rate of 1 mL/min and was monitored at 215 nm. The identified AOPs were named as Wuchuanin-AOPs and showed by an arrow.

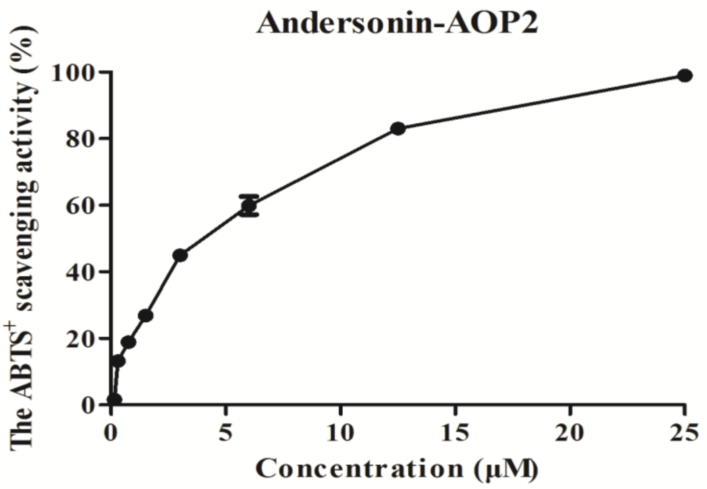

Figure S3. The dose-dependent ABTS^{+} scavenging activity of Andersonin-AOP2.

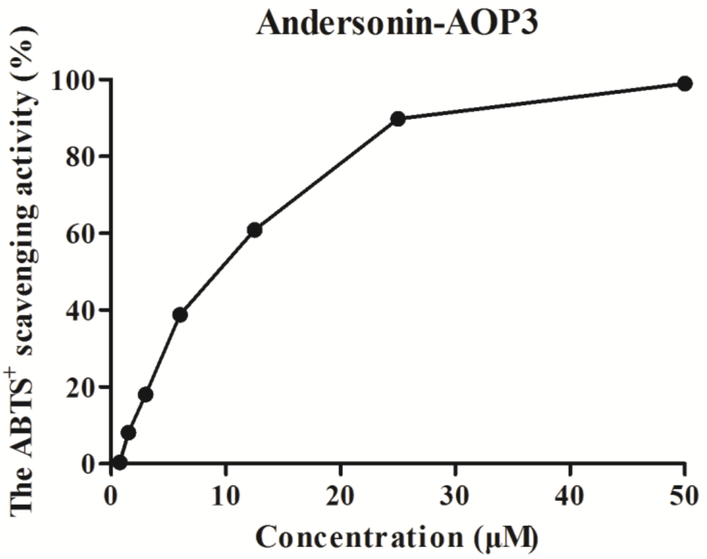

Figure S4. The dose-dependent ABTS^{+} scavenging activity of Andersonin-AOP3.

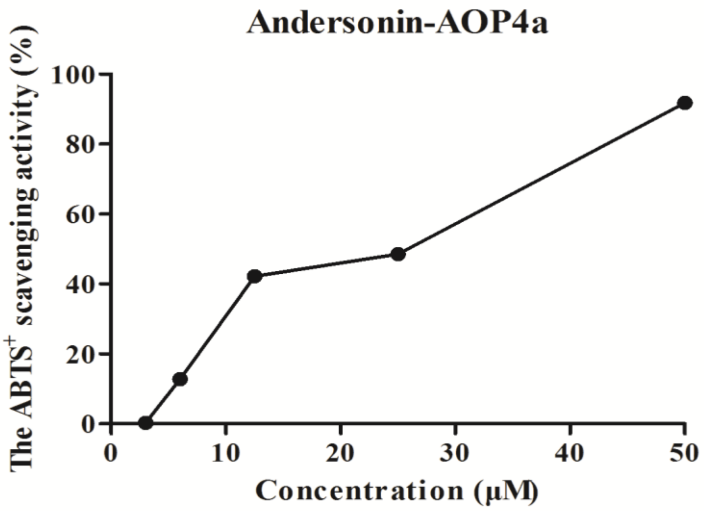

Figure S5. The dose-dependent ABTS$^+$ scavenging activity of Andersonin-AOP4a.

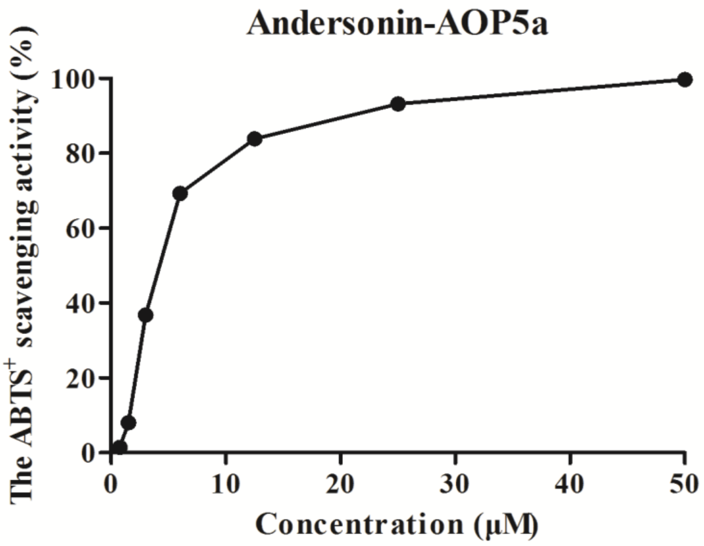

Figure S6. The dose-dependent ABTS$^+$ scavenging activity of Andersonin-AOP5a.

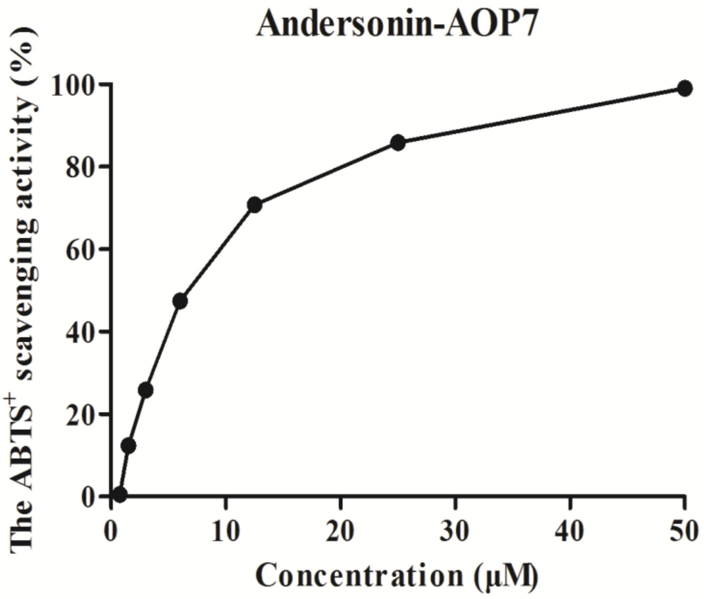

Figure S7. The dose-dependent ABTS$^+$ scavenging activity of Andersonin-AOP7.

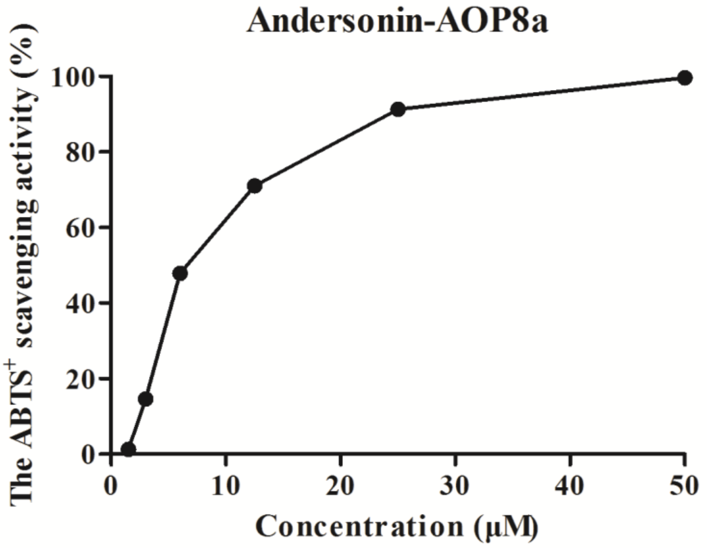

Figure S8. The dose-dependent ABTS$^+$ scavenging activity of Andersonin-AOP8a.

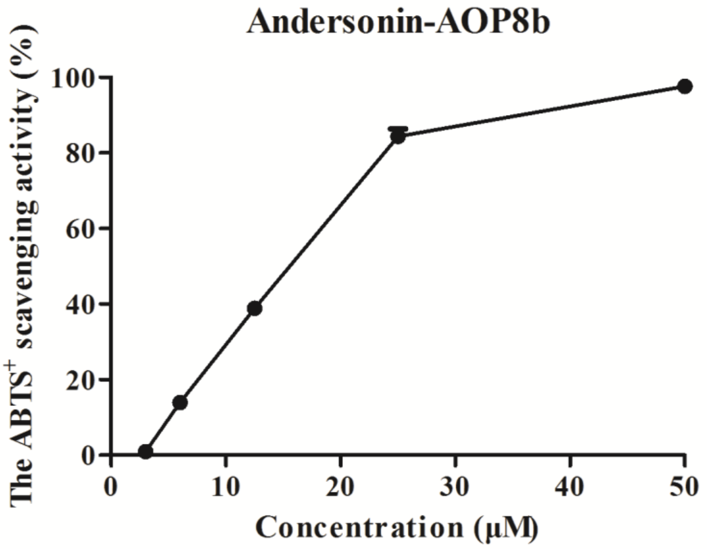

Figure S9. The dose-dependent ABTS$^+$ scavenging activity of Andersonin-AOP8b.

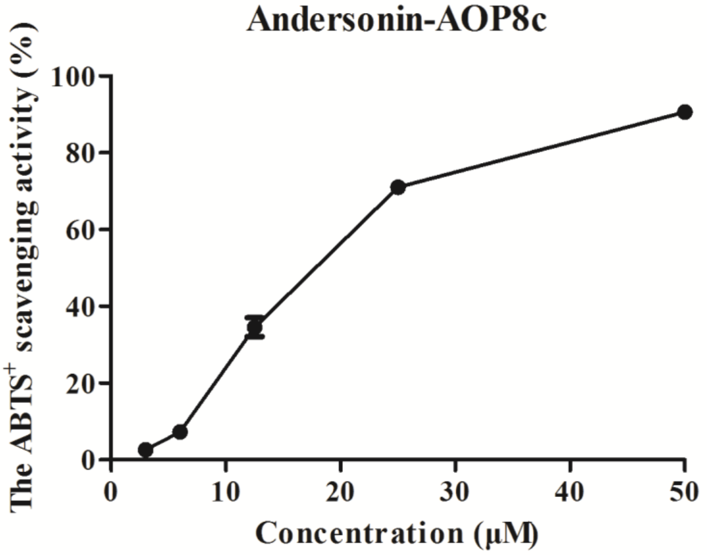

Figure S10. The dose-dependent ABTS$^+$ scavenging activity of Andersonin-AOP8c.

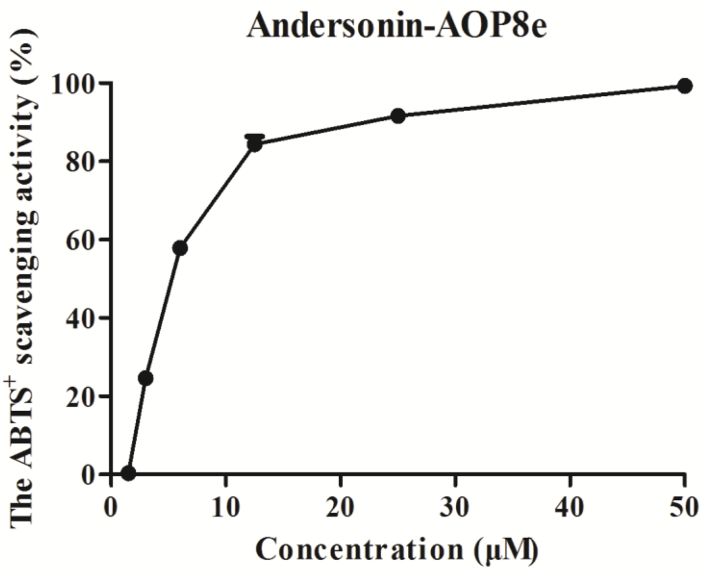

Figure S11. The dose-dependent ABTS+ scavenging activity of Andersonin-AOP8e.

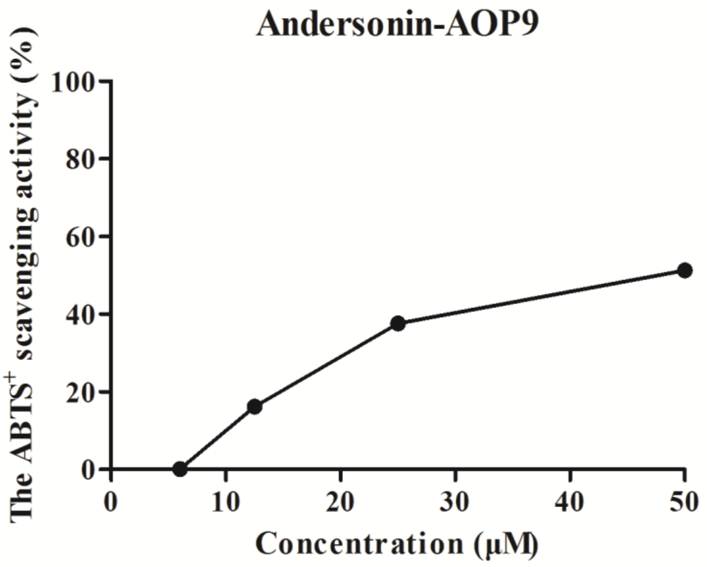

Figure S12. The dose-dependent ABTS+ scavenging activity of Andersonin-AOP9.

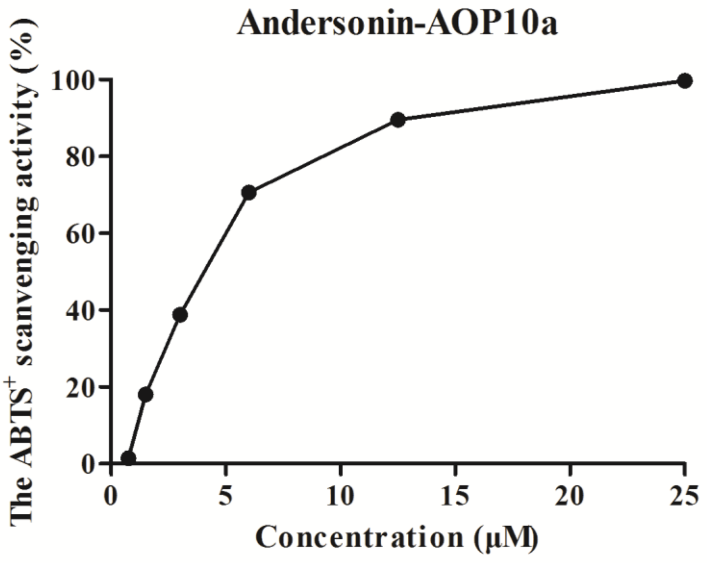

Figure S13. The dose-dependent ABTS+ scavenging activity of Andersonin-AOP10a.

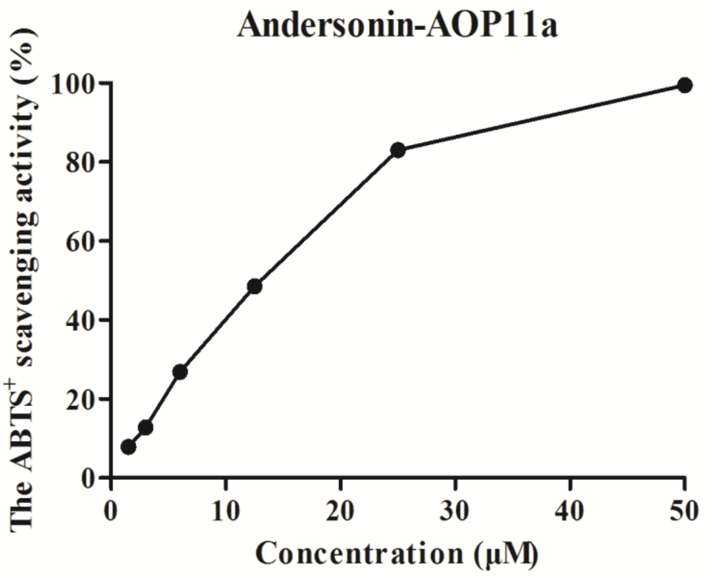

Figure S14. The dose-dependent ABTS$^+$ scavenging activity of Andersonin-AOP11a.

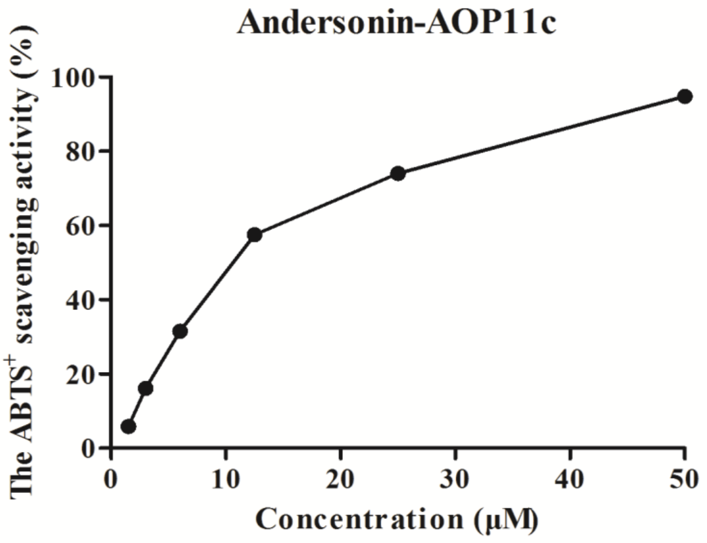

Figure S15. The dose-dependent ABTS$^+$ scavenging activity of Andersonin-AOP11c.

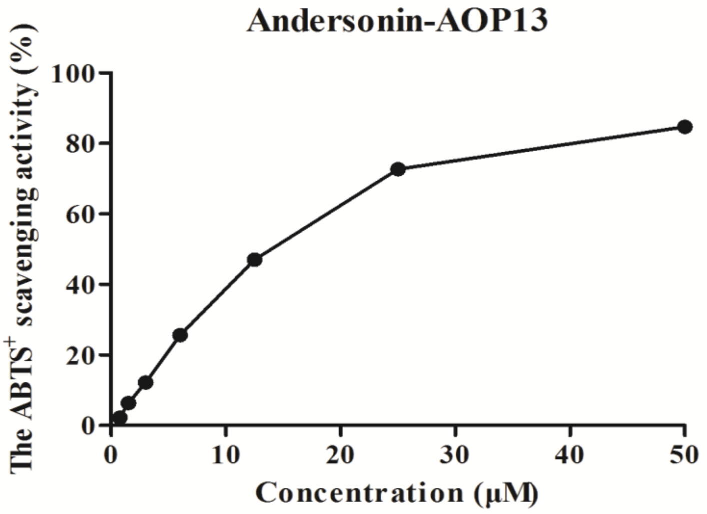

Figure S16. The dose-dependent ABTS$^+$ scavenging activity of Andersonin-AOP13.

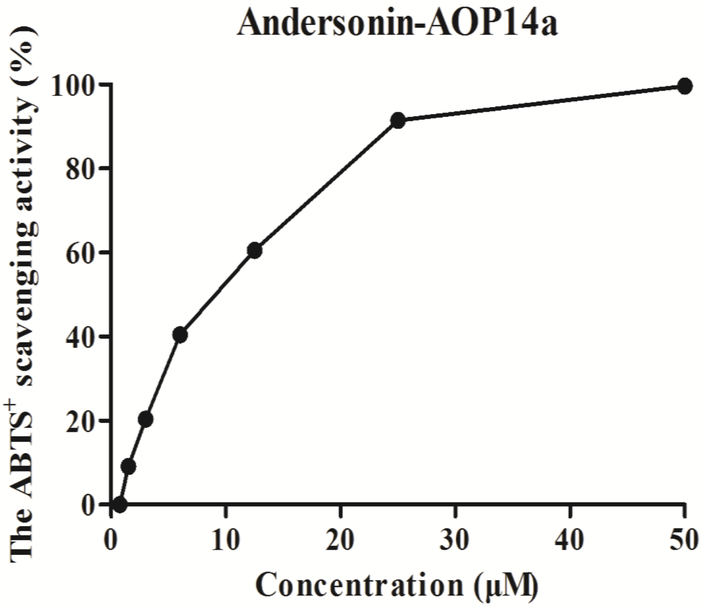

Figure S17. The dose-dependent ABTS$^+$ scavenging activity of Andersonin-AOP14a.

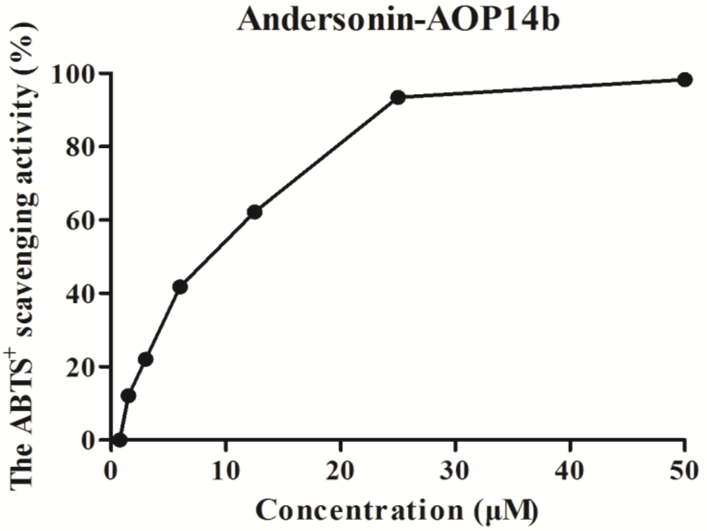

Figure S18. The dose-dependent ABTS$^+$ scavenging activity of Andersonin-AOP14b.

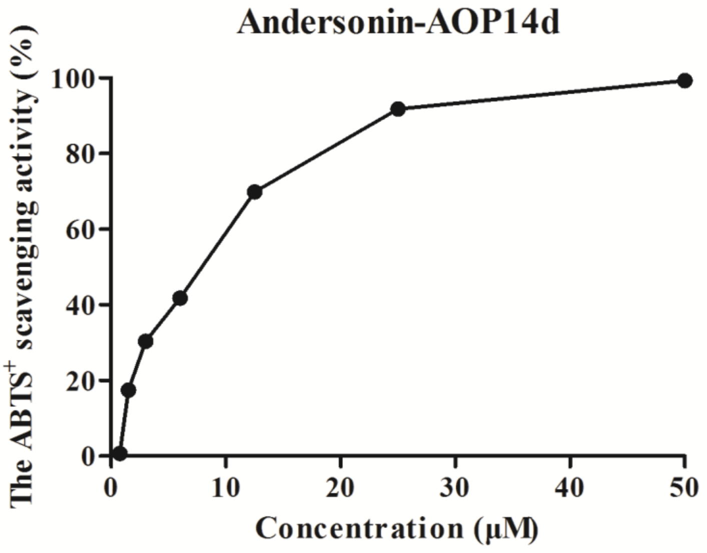

Figure S19. The dose-dependent ABTS$^+$ scavenging activity of Andersonin-AOP14d.

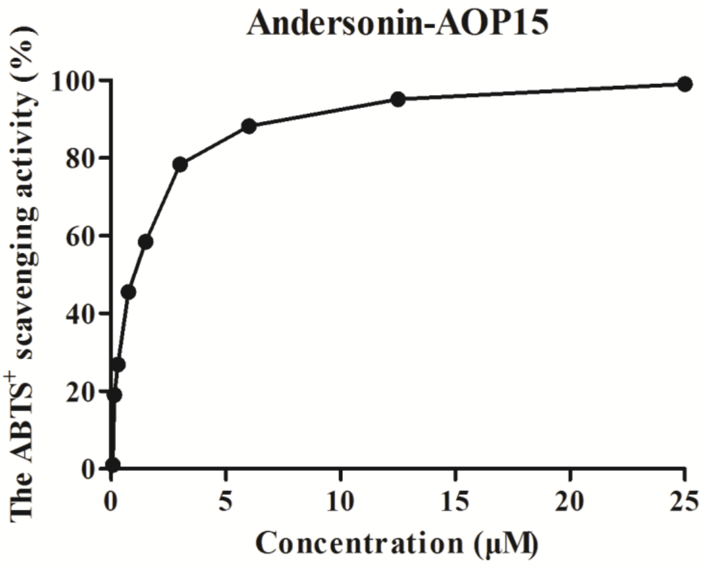

Figure S20. The dose-dependent ABTS$^+$ scavenging activity of Andersonin-AOP15.

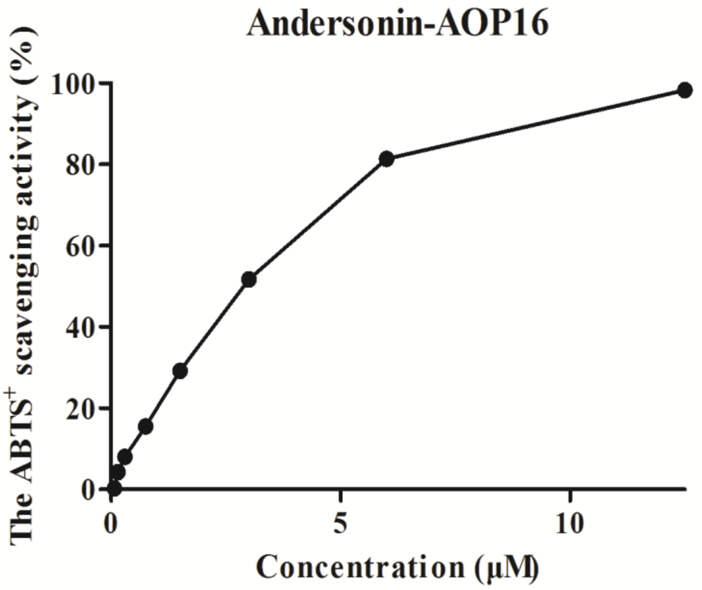

Figure S21. The dose-dependent ABTS$^+$ scavenging activity of Andersonin-AOP16.

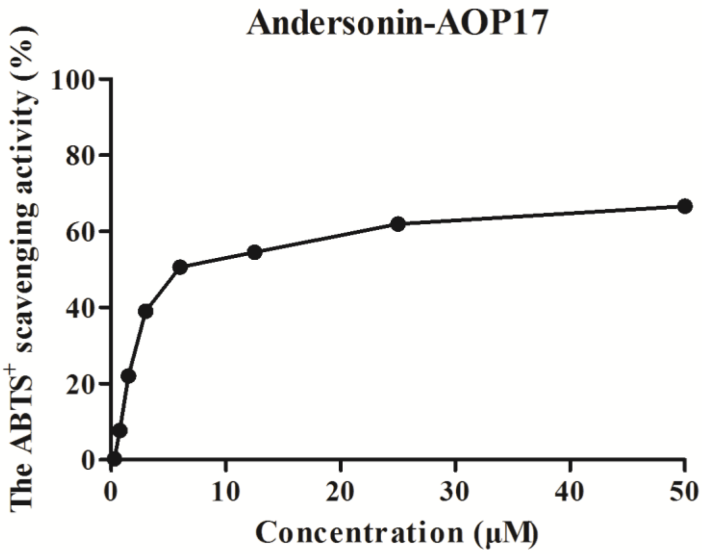

Figure S22. The dose-dependent ABTS$^+$ scavenging activity of Andersonin-AOP17.

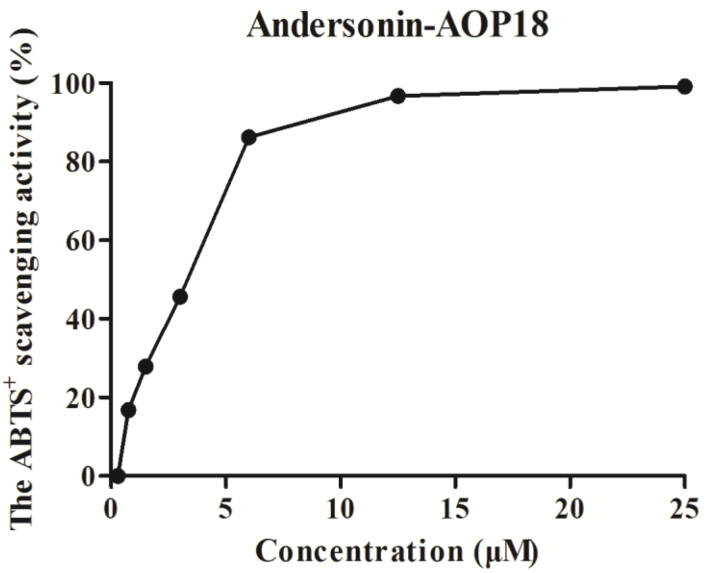

Figure S23. The dose-dependent ABTS$^+$ scavenging activity of Andersonin-AOP18.

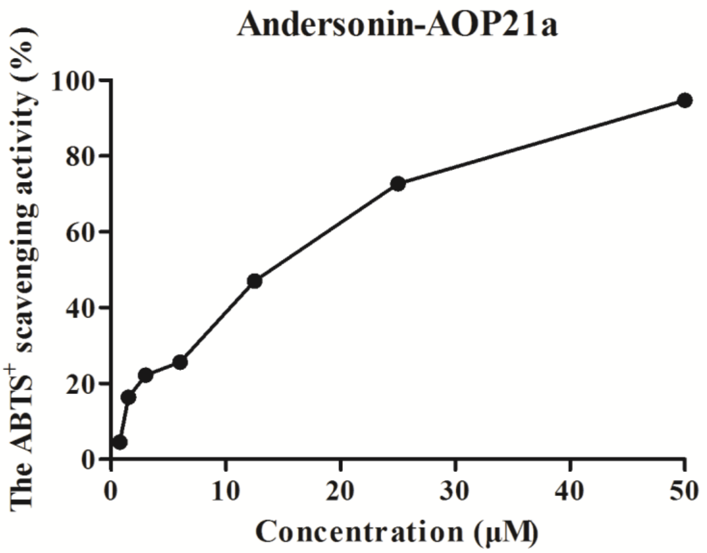

Figure S24. The dose-dependent ABTS$^+$ scavenging activity of Andersonin-AOP21a.

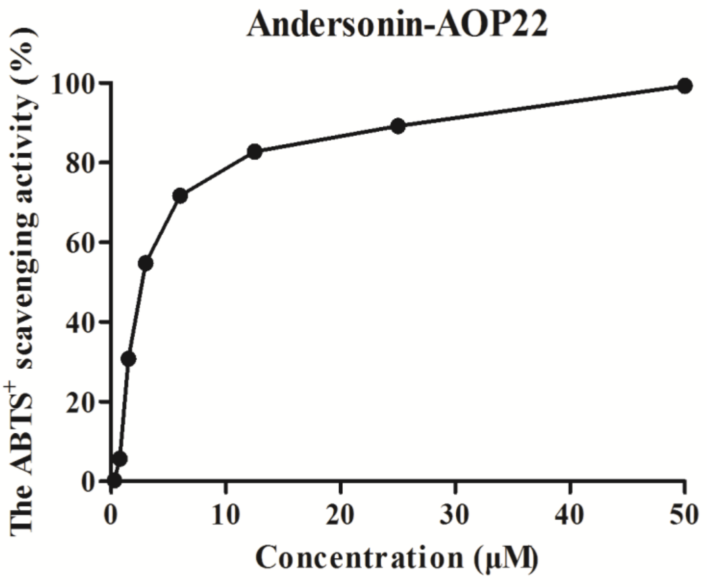

Figure S25 The dose-dependent ABTS$^+$ scavenging activity of Andersonin-AOP22.

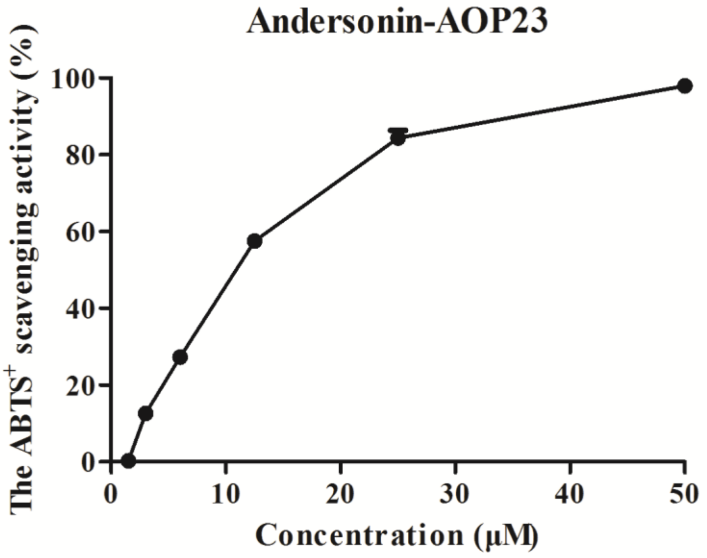

Figure S26. The dose-dependent ABTS+ scavenging activity of Andersonin-AOP23.

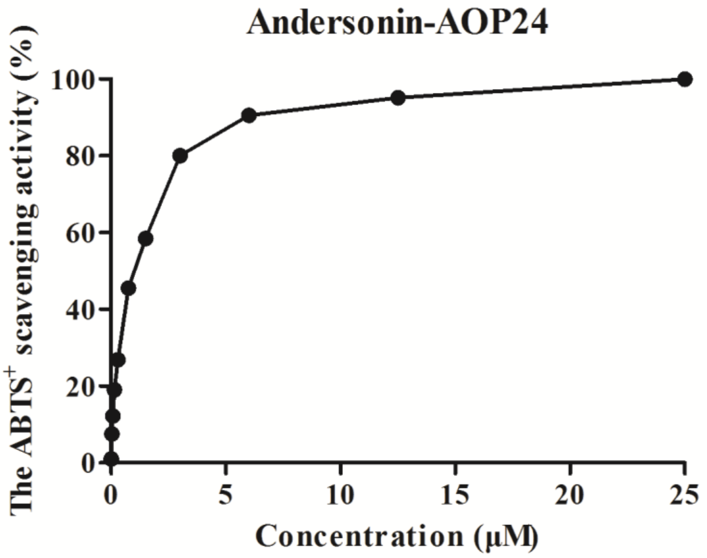

Figure S27. The dose-dependent ABTS+ scavenging activity of Andersonin-AOP24.

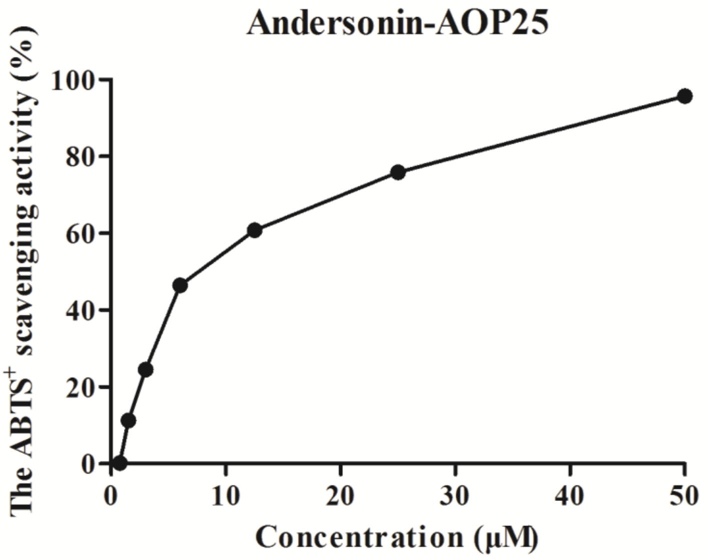

Figure S28. The dose-dependent ABTS+ scavenging activity of Andersonin-AOP25.

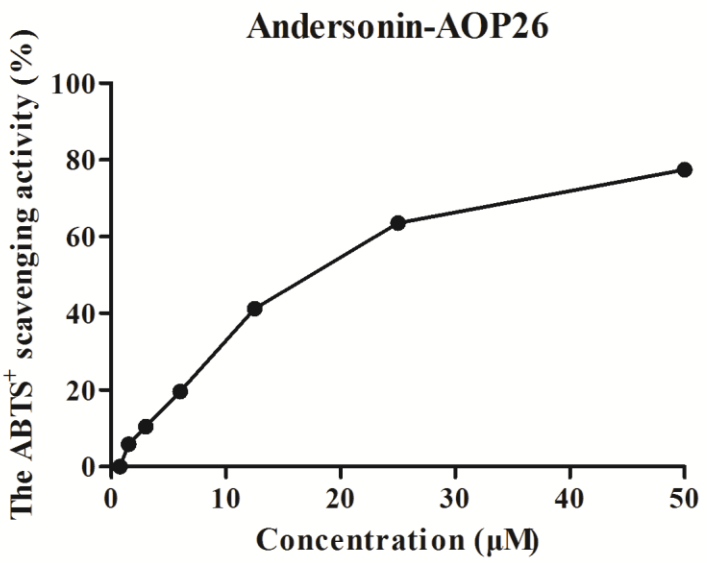

Figure S29. The dose-dependent ABTS$^+$ scavenging activity of Andersonin-AOP26.

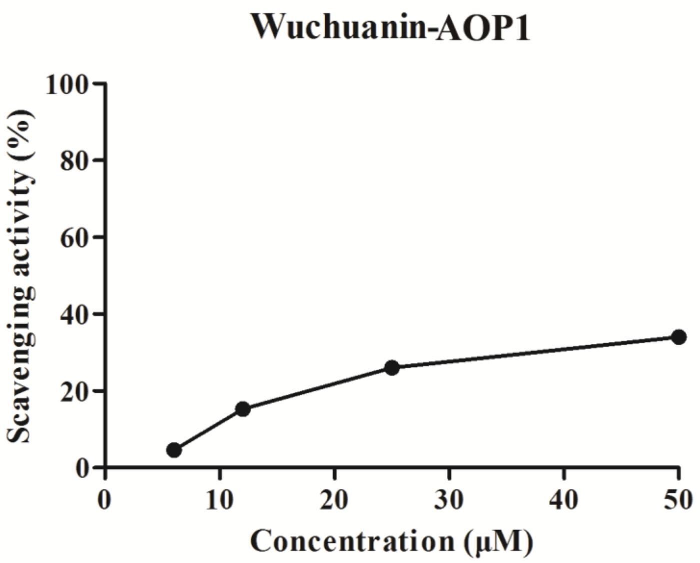

Figure S30. The dose-dependent ABTS$^+$ scavenging activity of Wuchuanin-AOP1.

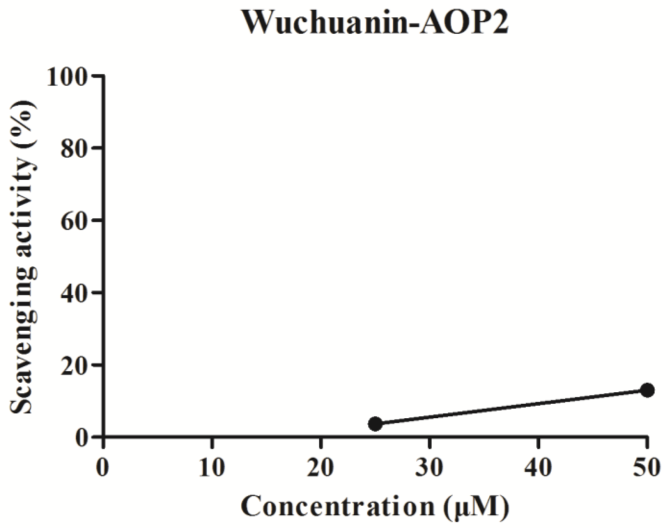

Figure S31 The dose-dependent ABTS$^+$ scavenging activity of Wuchuanin-AOP2.

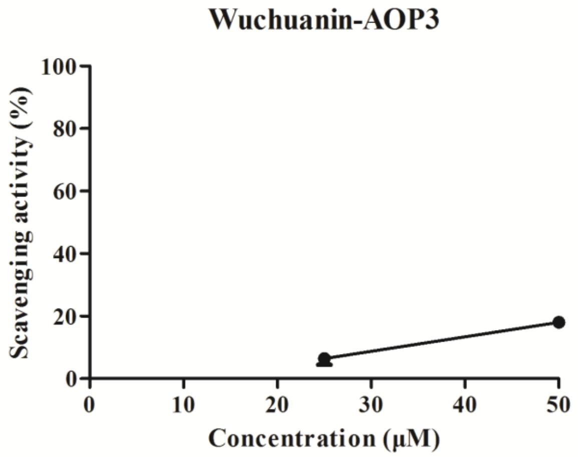

Figure S32. The dose-dependent ABTS$^+$ scavenging activity of Wuchuanin-AOP3

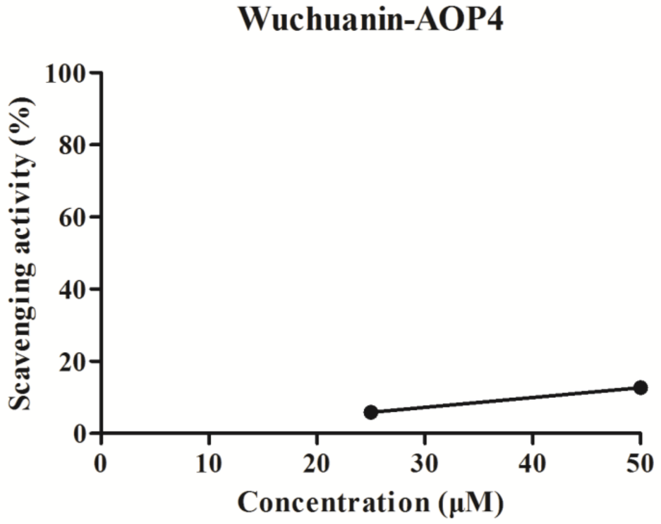

Figure S33. The dose-dependent ABTS$^+$ scavenging activity of Wuchuanin-AOP4
